# Epistemic Functions of Replicability in Experimental Sciences: Defending the Orthodox View

**DOI:** 10.1007/s10699-023-09901-4

**Published:** 2023-02-18

**Authors:** Michał Sikorski, Mattia Andreoletti

**Affiliations:** 1 Warsaw University of Technology, Warsaw, Poland; 2 Department of Health Sciences and Technology, ETH Zürich, Zürich, Switzerland

**Keywords:** Experimental Science, Replicability, Replication

## Abstract

Replicability is widely regarded as one of the defining features of science and its pursuit is one of the main postulates of meta-research, a discipline emerging in response to the replicability crisis. At the same time, replicability is typically treated with caution by philosophers of science. In this paper, we reassess the value of replicability from an epistemic perspective. We defend the orthodox view, according to which replications are always epistemically useful, against the more prudent view that claims that it is useful in very limited circumstances. Additionally, we argue that we can learn more about the original experiment and the limits of the discovered effect from replications at different levels. We hold that replicability is a crucial feature of experimental results and scientists should continue to strive to secure it.

## Introduction: It is Not all About Replication! Is it?

Replicability is widely considered to be one of the defining features of science by many methodologists (e.g., Munafò et al., 2017; Simons, 2014). Scientific results must be replicable to be reliable or trustworthy. At the same time, it is unknown to what extent actual scientific results are replicable. Some approximation is provided by large-scale replication projects, such as the Open Science Collaboration (2015) or Klein et al. (2013). The results of these studies were disappointing, with around 50% of conducted replication attempts being successful. This has convinced many that actual replicability rates in some disciplines, such as psychology, are too low. This low rate of replicability exemplifies the Replicability Crisis, which is believed to be problematic by most scientists (see e.g., Anvari & Lakens, 2018; Vazire, 2018; Open Science Collaboration, 2015) and some philosophers (see e.g., Romero, 2017; Hudson, 2021a).

Two general sentiments are present in the literature devoted to the Replicability Crisis. On the one hand, methodologists and meta-researchers share a general excitement. They view the crisis as a period of intense examination and improvement of science (Vazire, 2018). It is bringing about many salient changes in both methodology and the social structure of scientific practice, aimed at promoting replication efforts and increasing replicability of scientific results. On the other hand, some influential philosophers of science have been more cautious (see e.g., Guttinger, 2020; Feest, 2019; Leonelli, 2018; Andreoletti & Teira, 2016; Norton, 2015; Irvine, 2021).^1^ They have contested the "power" of replicability as a guiding principle for delivering reliable scientific results. This tension reveals a fracture between traditional philosophy of science, which has been trying for decades to provide an idealized theory of scientific rationality, and the new field of meta-research (Ioannidis, 2018) emerging in response to the replicability crisis. If philosophers are right that it is not all about replication, then why is replicability still considered a basic tenet of science^2^? And why do both scientists and scientific institutions invest so much cognitive and financial effort to pursue it? Are they wrong? Are they wasting time and resources?

In our paper, we want to fill the gap between these two perspectives by reassessing the role of replicability from an epistemological perspective. We start by presenting a standard taxonomy of replicability, ranging from direct replications, whose experimental designs are as similar to the original experiments as possible, to conceptual replications, which test the same hypothesis but with completely different experiments. Then, we discuss three philosophical arguments against replication and replicability. We present the orthodox view highlighting the usefulness of replicability and discuss how to get the most from replication and how to interpret a failed replication. We then respond to the critics of replicability. We will build on the orthodox view and show that the arguments challenged against replicability in philosophy fall short, partly because they are based on a narrow understanding of the concept. Finally, we deal with the science-policy consequences of our account.

## The Many Faces of Replicability

Answering the question "What is replicability?" is paramount to any informed discussion of the concept. Yet, the terminology concerning replicability and replication is very confusing, as the term replication is used to encompass a wide range of activities. Therefore, it will be useful to take a step back and consider two pre-theoretical features of replicability and reconstruct how the notion is commonly used in the literature.^3^

First, let us discuss the modal component of the concept. As most of the words in English ending with the suffix “-ility’’, replicability describes an ability rather than a categorical property. Other examples are nouns such as fragility or flexibility. Just as other abilities, replicability can be truthfully predicated about an object even if it was never realized or tested. For example, a vase can be fragile without ever being broken. Similarly, a result can be replicable without ever being replicated. It is enough for an experiment to be replicable if, for example, it would give the same result when conducted the second time.

Second, replicability is the ability to be replicated. To replicate means to copy, duplicate or repeat. Usually, the word "replicate" is used in contexts that suggest that the object or state created during the replication must be in some way similar to the replicated one. For example, when we say that the DNA was replicated, we claim that there is a high degree of similarity between the input and output of the replication. We would not call a process a replication if its output is not sufficiently similar to its input. For example, we would likely not call an abstract painting a replication of whatever it depicts.

What do the above considerations mean for the replicability predicated on scientific experiments?^4^ An experiment is replicable if, in proper conditions, it will be replicated. This formulation combines both features of replicability. However, there is still plenty of ambiguity in this formulation, and we will try to untangle it.

First of all, we can just mean that it is possible to repeat the experiment, for example, because we know enough about how it was originally performed. Alternatively, replicability may mean something more substantial, namely that we can repeat the experiment and, if we do it, the results will be substantially similar in some respects. Replicability is used in both basic senses (for the first see e.g., Schwab et al., 2000; for the second see Goodman et al., 2016). Nonetheless, the second sense is both more interesting for our purposes and more popular in the scientific literature, so we will focus on it. So, an experiment is replicable if we can conduct a similar experiment and the result of this replication would be in some relevant respect similar to the result of the original experiment. Once again, this formulation is ambiguous. It does not specify which aspect(s) of the design should be similar to the original experiment and what exactly is meant by ‘similar’. Experiments are complex and their designs involve many elements. Following LeBel et al. (2018), we can list contextual variables, physical setting, procedural details, stimuli, and the population used, as well as who is responsible for conducting the experiment, as key aspects of a study. For each aspect of the original study, the analogous aspect of a replication can be the same, completely different, or somewhere in-between.

Due to this ambiguity, there are many distinct notions of replicability. These can be classified according to how methodologically similar the replication study is to the original experiment. Direct replications are designed to be as similar as possible to the original experiment, while less similar replications may be conducted by different scientists and/or use a design that differs from the original in some important respect. Finally, a completely different experiment may be used to support the original hypothesis, in what is usually called a conceptual replication or triangulation (see e.g., Nosek & Errington, 2017). It is also not clear what exactly is meant by the result of a replication being similar to the result of the original experiment. There are many ways to conceptualize this similarity, such as the similarity of effect sizes or the result of a meta-analysis combining original and replication effects.

Based on these ideas about replication, we can attempt to define replicability. Replicability can be assessed based on a single replication attempt, in which case a study is considered replicable if the replication attempt is successful, and not replicable otherwise. Alternatively, replicability can be assessed by performing multiple replications and determining if the ratio of successful replications to unsuccessful ones is above a specific threshold.

The ambiguity inherent in the concept of replication also carries over to the concept of replicability. For both concepts, many possible specifications demand different levels of similarities in designs and results. In the rest of the paper, we will focus mainly on two of the mentioned dimensions of replication: its success and the similarity of the study design to the design of the original experiment. In order to categorize a replication attempt, we will need to know the hypothesis that is the target of the replication and the experiment to which it is being compared. Then, we can use one of the two ways of defining replicability to determine if the result of the experiment in question is replicable or not.

## Arguments Against Replicability: The New Localism

Is replication a general epistemically useful activity? Is replicability an indicator of research quality? Surprisingly, some philosophers of science tend to answer these questions negatively. As Guttinger (2020) has recently noted, “several authors have argued (a) that issues with replicability are not a general problem in science and (b) that the ideal of replicability does not universally apply to all disciplines” (p. 2). Guttinger describes this trend in philosophy of science as a “new localism” (p. 6) and implies that the epistemic value of replicability is heavily context-dependent.

For instance, John Norton has argued that the principle of replicability is not supported by a correspondingly universal principle in inductive logic. He presented examples of replicated results that were not accepted by a scientific community and results that were not replicated but were accepted anyway. Therefore, successful replication of experiments is not a good evidential guide. As he put it, "the idea of reproducibility is merely a gloss on inferences that are quite specific to the case at hand and dependent essentially on background assumptions. […] Understood as a formal principle, reproducibility gives us no real guidance." (Norton, 2015 p. 241). In his view, replicability understood as a general principle, does not warrant any inductive inference. Rather, Norton claims that inductive inferences are warranted only insofar as there is an agreement on the particular facts (background assumptions) prevailing in each experiment.

Along the same lines, Sabina Leonelli (focusing on what she calls “direct reproducibility”) holds that the pursuit of replicability works well as an epistemic principle only in those fields in which there is high standardization of methods and materials and a very high degree of control over experimental conditions (e.g., software development). In other research fields, replicability is "neither fruitful nor desirable" (Leonelli, 2018) since it can divert attention from a more critical approach to the evaluation of evidence. According to Leonelli, the defenders of replicability focus mostly on the use of it as the best research strategy "to achieve inter-subjectively reliable outcomes” (p. 132), and she argues that such an aim can be achieved even in the absence of replication. In scientific research, there are many legitimate fields of inquiry where replicability has little or no role, but scientists can nonetheless agree on the results. This holds especially in those research fields where we have both limited control over "environmental variability" and limited reliability of statistics as an inferential tool, such as research on experimental organisms or archaeology. In this case, Leonelli argues that direct and indirect replicability are not very helpful. There are also fields, namely observational research such as case reports or ethnographic work, where replicability seems a strange concept. In these cases, replicability has very little meaning and researchers try to achieve robustness of results by employing other research strategies. For these reasons, she openly disagrees with the use of reproducibility as a “regulatory ideal for science” (p. 138).^5^

More recently, Uljiana Feest (2019) made a further case against replications in psychological research. According to Feest, replication is not “as central to experimental practice as it is sometimes taken to be'' (p. 901). In fact, she argues that both direct and conceptual replication have very limited epistemic value.^6^ In regards to direct replication, assuming that it is achievable, it can help researchers rule out random errors, but it fails to address systematic errors. This is because any experiment, including replications, involves some sort of "individuation judgments," i.e., researchers' judgments on experimental design and context. These judgments rely on tacit assumptions about the relevance of variables that should be controlled within experiments. For example, one may think that the temperature in the lab is irrelevant to the results and ignore it in the replication attempt, while instead, it may be very relevant to the effect. On the other hand, if there is bias in the original experiment's design, the direct replication will replicate that bias as well. Neither successful nor failed replications can tell us much about unknown confounding variables. As Feest notes, "it is obvious that there is always a danger of systematic error because there is always a possibility of overlooked confounding variables" (p. 902). And this problem also affects conceptual replications. In fact, researchers usually cannot correctly identify and describe dependent and independent variables for a given effect, which are fundamental to defining the scope of the effect one is trying to replicate. Conceptual replication presupposes a good understanding of the relevant concepts, which researchers often lack.

Finally, Feest argues that experiments in psychology are typically exploratory in nature, and therefore, it is not very useful to replicate their results. Instead of insisting on replications, Feest suggests that "productive research should (and frequently does) proceed by exploring, and experimentally testing, hypotheses about possible systematic errors in experiments" (p. 904). As a result, it seems that replications, either direct or conceptual, can add little evidence and are less useful and important than is widely assumed in the debate on the replicability crisis.

A similar argument against replicability was presented in (Irvine, 2021). She argues that both conceptual and direct replication do not provide significant theoretical insights that can be later used in the construction of new psychological theories. The direct replications, according to Irvine, can at best show that a procedure (e.g., experiment) that produced the original result is repeatable. Mere repeatability is not very useful in theory construction. Stronger conclusions from the results of direct replication require the presence of a well-developed background theory, which, according to Irvine, is typically not available in psychology. It is similar in the case of conceptual replications. Their results are informative only when we know a lot about the validity of measurements used in both experiments etc. Consequently, Irvine argues that replications conducted in fields that lack well-developed theories, like psychology, should be understood as exploratory studies.^7^ The results of such studies can be used to inform theory development.

Before addressing the philosophical criticisms in detail, in what follows we present a positive view of replicability by arguing that replicating experiments is (always) an epistemically beneficial activity. Such an approach to replicability is dominant in the contemporary methodology of science and meta-research. And this account is instrumental to our responses to the critics of replicability.

## The Orthodox View: Replications are *Always* Epistemically Beneficial

As we have seen, replicating an experiment means conducting a similar experiment with the goal of obtaining a similar result. A replication, like any experiment, provides additional evidence concerning a given hypothesis. Regardless of the outcome, replications improve our understanding by providing additional evidence. If successful, it supports the hypothesis, otherwise, it undermines the evidence provided by the original experiment and contributes to the falsification of the tested hypothesis. Replications from different levels can provide different information, beyond just providing additional evidence for the effect. They may be used to test the limits of the original effect or if it depends on some of the assumptions of the original experiment. Results of interviews with prominent scientists suggest that replications are often conducted mostly for that secondary evidence (Peterson & Panofsky, 2020). These replications are called integrative replications and are contrasted with diagnostic replications, conducted primarily to test the original effect.

If a *direct replication* is successful, it shows that it is very unlikely that the positive results of the first experiment were due to a statistical accident. Direct replication is especially useful if the original experiment is underpowered and therefore likely to deliver an inflated effect (see e.g., Ioannidis, 2005, 2008; Pereira & Ioannidis, 2011). If the direct replication fails, then given that the designs of both experiments are very similar, the failure is also informative.^8^ Multiple failed replications in the case of a well-powered original study may suggest that questionable research practices were used. Generally, original studies are usually more suspected of being methodologically flawed because they are typically not pre-registered, while replication studies often are (see Nosek et al., 2018, 2019; Chambers, 2012).

*Conceptual replication* lies on the opposite end of the spectrum from direct replication. If successful, it provides independent evidence of the hypothesis, showing that it can be supported without relying on the assumptions and theory of the first experiment.^9^ It can be used to test the limits of the effect, for example, by testing if the effect is present in a different population (see e.g., Van Dongen et al., 2020). Evidence obtained from two independent sources is stronger than evidence obtained twice from the same source.^10^ If a conceptual replication fails, it does not provide much insight because there are many possible reasons for failure, such as a statistical accident or questionable research practices. Additionally, the difference in the designs of both experiments may cause a difference in results. It may be that one of the assumptions on which one of the experiments is based is false, making the experiment unreliable.

Finally, some types of replications *fall somewhere in between* conceptual and direct replications. The similarities and differences between the replication and the original study determine what can be learned from the replication. If both experiments are relatively similar, a failed replication can point to a potential problem with the population used in the original study. A successful replication confirms the original hypothesis and suggests that it generalizes beyond the sample used in the original study. Additionally, replications that differ from the original study in any aspect of its experimental design can be used to test if the change alters the result. For example, it may be the case that our hypothesis is that a given substance is toxic for all mammals and the original experiment was conducted with rats. If we have doubts about how representative rats are for mammals, it may be beneficial to conduct a similar experiment on different experimental animal models, for example, rabbits or monkeys. A failed replication points to a potential problem with one of the used populations. For example, one of them may be too sensitive or not sensitive enough (see e.g., Wilholt, 2009). If the replication is successful, it corroborates the original hypothesis and suggests that it generalizes beyond the sample used in the original study. Similarly, we can use a replication that differs from the original study in any aspect of its experimental design to test if this change does alter the result.

As we have seen, replication can fulfill two traditional functions in philosophy of science: corroboration and falsification. A successful replication is a step towards corroborating the re-tested hypothesis, while an unsuccessful one is a step towards falsifying it. Replication can also fulfill two additional functions. Firstly, it has epistemic benefits that go beyond just gathering additional evidence. These benefits stem from the fact that during replication, the designs of the original study and replication are explicitly compared, which is not present in concepts of corroboration and falsification. Secondly, the notion of replicability is not susceptible to some of the criticisms made against traditional notions. For example, as pointed out by Lakatos (1978), it is unclear what observation—if any—constitutes a successful corroboration or falsification. Many, if not all observations that *prima facie* constitutes a falsification of a given hypothesis can be explained in a way that saves the hypothesis. This is not the case for failed replications. Replication relates two experimental results, rather than experimental results and hypotheses, making it easier to define clear conditions for success. The right measure of replicability is still controversial. The Open Science Collaboration (2015) uses five different measures of successful replication, yet all used measures give similar results. Unlike in the case of falsification, there seems to be no reason preventing us from coming up with reliable success conditions for replication.

### Should We Care About Ir-replicability? And How to Interpret a Failed Replication*?*

In light of the above, it is clear that replication is an epistemically useful activity. But what about replicability? Should scientists strive for it? Do unsuccessful replications and ir-replicability tell us anything about the quality of the original experiment and the truth of the tested hypothesis?

It is clear that if a study is not replicable, it means that the original result and tested hypothesis cannot be reliably supported by new evidence. This suggests that replicability is expected when the experiment is well-designed and its hypothesis is true. Therefore, if we show that a study is not replicable by conducting many well-designed and executed but unsuccessful replications (we are using an operationalization of replicability from Klein et al. 2013 here), it strongly suggests that its hypothesis is false. Alternatively, it may be the case that the results depend on the specifics of the original experimental setup and therefore cannot be replicated. Such cases are sometimes used to argue that replicability of scientific results is not a realistic requirement for some disciplines (Feest, 2019). However, such results, common in social psychology, even if literally true in extremely narrow circumstances, are typically both overstated and too local to be ever useful (Yarkoni, 2019). In light of this, it seems that the majority of meta-scientists and methodologists are right, and replicability is a crucial feature of a scientific result. Irreplicable results are false or, in the best-case scenario, ungeneralizable, and therefore can hardly be considered to be scientifically valuable. Consequently, given that the only sure way to demonstrate replicability is to replicate the result, in any case in which there are justified doubts concerning replicability, replication should be attempted.

The final question to discuss is how much we can learn from a single replication. Firstly, a failed replication by itself does not tell us much about the quality of the original study. This is because there are many possible causes for the failure of replication, and problems in the original studies are just one of them. Firstly, the result of the original study may be a false positive despite the experiment being of good quality. Secondly, the differences between the designs of the two experiments introduce a sampling error and other random errors which can result in different results (see e.g., Gilbert et al., 2016). No two experiments can be methodologically identical, so such errors are unavoidable (see e.g., Anderson et al., 2016). Finally, both the original experiment and replication may be flawed in some way. For example, one of the experiments may be underpowered, questionable practices may be used, or some of the results may not be reported. Because of all these factors, even if we assume that the quality of all the experiments in a given field is high, the expected replication rates are rather low. For example, the authors of (Open Science Collaboration, 2015) expected 78.5% of replicability using one of the described measures (sCI) (see (Open Science Collaboration, 2015) supplementary information, pp. 56 and 76; https://osf.io/k9rnd).

The presence of possible alternative causes of replication failure was used to explain low replicability (e.g., Gilbert et al., 2016) or even to argue that we cannot infer anything about the quality of the study from a failed replication (Feest, 2019; Leonelli, 2018). Scientists seem to be concerned about failed replications, and the expected rates of replicability, despite being rather low, are still much higher than estimates obtained in large-scale replication projects. This suggests that many experiments are of sub-optimal quality, and a failed replication can be a step toward identifying such experiments. We believe that a failed replication should at least be interpreted as a red flag, as it raises doubts about the quality and truth of the original study's results. It should be a starting point for a discussion about the relevance of the failed replication for the original study, with a focus on the quality of the replication rather than the similarity of the experimental designs. As we have seen, a replication can provide evidence for or against a given result regardless of how different it is from the original experiment. As long as the design of the replication is reliable in testing the hypothesis in question, its results are relevant for the original experiment. Additionally, we have reasons to believe that replications are more likely to be methodologically sound than the original experiments. They are less likely to suffer from publication bias, as they are often pre-registered, meaning all results are disclosed. For example, a special type of replication called Registered Reports (Chambers, 2012) are accepted before the experiment is performed, so there is no publication bias, and the use of questionable research practices is restricted (see https://cos.io/rr/).

To summarize, in this section, we have argued that replication is epistemically beneficial. It not only provides evidence concerning the tested hypothesis but also information about the design of the original experiment. As we have shown, what we can learn depends on the result of the replication and how similar its design is to the design of the original study. Therefore, the level of replication should align with the interests at hand. Additionally, we have stated that a failure to replicate should be considered a red flag. Furthermore, when combined with other reasons to believe that the replication is methodologically superior, such as lack of pre-registration in the original study, it should be treated as defeasible evidence for the low quality of the original experiment. With each failed attempt to replicate, we gain additional evidence against the result of the original study.

## Defending Orthodox View

How does the criticism of the reliability and replicability described in Sect. 3 align with the traditional view that supports the epistemic usefulness of replication? As we have seen, the main point of both Leonelli and Norton was that there are cases in which replicability is difficult (or even impossible) to achieve, or not epistemically beneficial, and therefore, we should not generally prioritize replication and replicability. We agree with the premise of this argument, but we do not believe that the conclusion follows. There are indeed cases where a successful replication does not necessarily indicate the truth or fails to persuade interested scientists (such as in the case of successful but unconvincing replications of a study supporting the healing power of prayer discussed by Norton, 2015). This is not surprising. No statistical experiment, including replications, is perfectly reliable, and therefore, we can never rule out an incorrect result with absolute certainty. It is even less surprising that some replications are not persuasive. Experiments are not only fallible but also complex, and therefore, there are many possible causes of negative results that can be used to explain away the failed replication.

On the other hand, if we judge the replicability of a result on the basis of multiple replications then it seems that, contrary to Norton, we have a universal reason to value its replicability. A result that is not replicable in this sense (will not be supported by a significant majority of conducted replications) is not able to account for new evidence. This, in turn, means that as soon as the replications in question are conducted, the result ceases to be empirically adequate. If one cares about the empirical adequacy of the results, it seems clear that one should also strive to preserve this adequacy in the future. In light of this, they should strive for replicability. If we understand replicability in this way, and replication as an ongoing iterative process composed of multiple replications, then we have a universal reason to care for replicability and distrust irreplicable results. They will cease to be empirically adequate when further tested. This requirement of replicability makes replication necessary, even in light of the deficiencies of single replications. As far as we know, there is no other way to ensure replicability of a given result than successfully replicating it multiple times.

Similarly, as Leonelli notes, it may be the case that in some non-experimental scientific disciplines like archaeology, it is not clear how or even if the results can be replicated. But this does not imply that in the disciplines in which replicability is well understood, it should not be pursued or that it is overall any less epistemically valuable. Leonelli further argues that even in some non-standardized experimental settings, such as animal model research, direct replicability is nearly impossible to achieve. But this claim does not square with experimental practice. As Guttinger (2020) notes: “the way in which researchers in animal model research deal with the problem of plasticity and historicity shows that they don’t abandon the ideal of replicability when standardization and control become problematic” (p 12). Indeed, even in those contexts, replication remains a guiding principle of experimental design, as most experiments are done in triplicates.

Now, let's move to Feest's argument. As we have seen, she claimed that neither direct nor conceptual replication is particularly epistemically useful in experimental psychology. According to her, we do not learn much from the results of direct replications. Indeed, we do not learn much from the positive result of a direct replication in case we have high confidence in the original result. Given that we had confidence in the result in the first place, a successful replication will provide us with negligible evidence. However, the results of replication projects and other meta-scientific studies strongly suggest that in general, in the case of psychological results, such confidence is not warranted. Neither classical nor recent psychological results scored well in large-scale replication projects (see for example Klein et al. 2013 or the Open Science Collaboration, 2015) and we have many reasons to believe that the reliability of psychological results is much lower than it is typically believed. If one is somehow unsure about a given result, an attitude that seems to be justified about most psychological results, then additional evidence in the form of a successful replication is clearly epistemically useful. At the same time, we learn a lot from an unsuccessful direct replication. Such results, despite similar methodological designs of the experiments, are not always easy to interpret but, as we argued, they constitute prima facie evidence that the original result is false. For example, in the case of results presented in the Open Science Collaboration, (2015), even if we assume that all results were well established and therefore, we did not benefit substantially from successful replications, there is still 50% of surprising unsuccessful direct replications. They were useful not only in uncovering the state of psychology but also in providing some reasons to be suspicious about each result that failed to replicate. In light of that, it seems that the direct replications are epistemically useful.

What about conceptual replications? The main criticism challenged by Feest against it is that because of substantial differences between an original experiment and a conceptual replication, it is extremely hard or even impossible to interpret a possible difference in results. Scientists typically do not know enough about the dependent and independent variables and confounders. Therefore, we can never know what factor caused the difference in results and therefore it is not clear what, if any, conclusion we can draw from such results. In principle, it is possible to test the relevance of a given factor in a second experiment, but this experiment involves similar problems which lead to experimental regress. The ‘experimenter’s regress' is definitely a problem for interpreting replications (especially conceptual ones), but it is also a conundrum for interpreting the results of any experiment. We never know if one of the many factors present during the experiment is a confounding variable (see e.g., Collins, 2016). Analogous skeptical arguments were championed against the feasibility of other epistemic processes like perception (see e.g., Godin & Gingras, 2002). Given that all those arguments are analogous, it seems that if one is inclined to reject the epistemic value of conceptual replications on this ground (as done by Feest) then she should reject the epistemic value of experiments or even any epistemic processes like perception. This would lead directly to a general and untenable skepticism. At the same time, practicing scientists are typically not paralyzed by the prospect of their experiments falling into the experimenter's regress. And there are also suggestions on how to avoid it, and convincing arguments explaining why it is not as serious a problem as it was initially believed (see e.g., Zuppone 2016). Moreover, as Hudson (2021b) rightly points out, the fact that replication requires some knowledge concerning the influence of environmental conditions on the effect does not imply that a scientist performing a replication under different conditions (or without testing how new conditions influence the effect) “begs the question". In fact, replications can be used to test the influence of such environmental conditions.

Concerning a similar argument against replications from (Irvine, 2021), it seems that the framing of the article misses the main reason why replicability is attractive for psychologists. The main role of replications is not to be a tool for theory construction, but to improve the justification of already-formed hypotheses or theories. As mentioned above, when a theory or hypothesis is proposed and tested, the results can be replicated in order to validate or undermine it. That is the main function of replication, and it fits fully in the context of justification. In light of that, it seems unfair to judge replication as a tool for theory development, which is the context of discovery. Moreover, contrary to Irvine’s main claim, it seems that replications can, or even have to, be used in domains in which well-developed theories are not yet present (e.g., psychology). For instance, Eronen and Bringmann (2021) argue that one of the reasons for the poor state of psychological theories is that we do not possess robust knowledge about psychological phenomena (see also Borsboom et al., 2021 or Haig, 2013). These phenomena are to be explained by psychological theories, therefore, if we do not know how our mind behaves, it is not clear what our psychological theories should explain. Nonetheless, replication studies were useful in uncovering which of the widely accepted psychological phenomena are real and which are not (see Eronen & Bringmann, 2021 for examples).

Finally, we would like to object to the idea presented in both (Feest, 2019) and (Irvine, 2021) that replications (or even the majority of psychological experiments) are, or can be, interpreted as exploratory studies. Explicitly exploratory experiments are infrequent (see e.g., Gelman, 2016). At the same time, the exploratory-confirmatory nature of an experiment is not a matter of interpretation. The methodology used in exploratory experiments differs significantly from that employed in confirmatory studies. For example, as described in (Wagenmakers, Borsboom, and van der Maas 2011), exploratory statistical analysis, during which subsets of the sample are excluded "in search for" a statistical significance, is acceptable in exploratory studies but not in confirmatory ones. Mixing the elements of the methodologies of both types of experiments is problematic and can lead to an overestimation of obtained results. Consequently, the authors insist that the nature (exploratory-confirmatory) of the study should be clearly stated. In light of that, it is clear that confirmatory experiments, which constitute the majority of psychological experiments, can't be just interpreted as exploratory. The same seems to be true about the majority of replications. So, the proposals championed by both authors, according to which replications should be interpreted or used as exploratory studies, are both revisionary and costly to implement.

In conclusion, contrary to critics, and in line with the orthodox view, we maintain that replicability is crucial for experimental results and that scientists should strive to make their results as replicable as possible. All types of replications (from direct to conceptual) provide us with additional evidence for a given result and are therefore epistemically useful. Focusing too narrowly on a single replication, as was done by Norton and Feest, will lead to finding cases in which replications will be uninformative or unconvincing. This is not unique to replication; it is now clear in meta-science that we typically do not learn much from a single experiment (see e.g., Ioannidis, 2005). We cannot rely on a single replication just as we cannot rely on any single experiment. These difficulties are ameliorated by multiplying replications. As long as those replications are of acceptable methodological quality, the combined results will converge toward the truth. In light of that, a result which is irreplicable, in the sense that it will not be supported by the totality of evidence collected in the original study and multiple replications, cannot be trusted. We do not claim that the ideal of replicability can be applied to all sciences. As argued by Leonelli (2018), in cases of some non-experimental sciences, replicating may be difficult or even impossible. Nevertheless, in the case of experimental science, there are compelling arguments to claim that only replicable results are scientifically valuable.

## Science Policy

The proponents of the prudent view contest, at least to some degree, the usefulness of replication. In line with that, they claim that scientists should not focus on replications. For example, Irvine (2021) argues that instead of replicating, scientists should focus on exploratory studies.

So far, we have argued that replication not only provides additional evidence for the tested hypothesis, but it can also reveal something about the methodological limitations of the original experiment or the generalizability of the result. Consequently, replications should be incentivized. This conclusion is consistent with recent calls in the literature to prioritize and promote replication projects (see e.g., Zwaan 2017, Coffman et al., 2017, or Romero, 2017). In the rest of the section, we will discuss what kind of replications should be preferred and which results to replicate.

### What Kind of Replication to Conduct?

As we have seen, replication not only provides additional evidence for the tested hypothesis, but it can also tell us something about the methodological limitations of the original experiment or the generalizability of the result. What we can learn from replication depends on how similar its design is to that of the original experiment. Therefore, which type of replicability is useful in each case depends on what exactly we want to gain by performing the replication. Conversely, a replication of each type may be all but useless in some contexts; for example, most likely we will not learn a lot from a (likely successful) direct replication of a well-established result.

Here, we briefly discuss which type of replication is optimal in each situation and how to get the most from replicating. Before we move to discuss the advantages and disadvantages of different levels of replications, it is important to mention the minimal requirements that need to be satisfied by every replication. Such minimal requirements were proposed in Janz and Freese (2021). The authors convincingly argued that all replication studies should be as transparent as possible (see also Hensel, 2020), and the reason for the selection of the replicated experiment should be clearly stated. Original authors should be encouraged to comment on the replication, and any such comments should be taken under consideration. Finally, the authors claim that binary judgments (replication was successful/unsuccessful) should be avoided in reporting the results of the replications. We are less sure about this last recommendation. Some of the discussed epistemic functions of replication depend on a clear and decisive statement of its results. For example, we do not know if the results of replication support the replicated result or suggest that it is a false positive if those results were not stated in binary terms. We believe that this is one of the reasons why both single-study replications and large-scale replication projects typically use binary judgments to clearly state their results.

If we want to make sure that the result of our experiment is not a statistical fluke, we just need to make sure that our experiment is directly replicable (see e.g., Andreoletti & Teira, 2016). If we want to show that our hypothesis can be supported by independent evidence, we should make sure that it is conceptually replicable (see e.g., Nosek & Errington, 2017). Finally, if somebody is unsure about some of the aspects of the design of the original experiment, they should show that the same result can be replicated through an experiment that does not depend on the problematic assumptions. In an ideal situation, one would like to conduct multiple replications on all levels. If enough of them were successful, our hypothesis is replicable in all the senses of replicability. In such a case, a scientist in question would have obtained all possible epistemic benefits which can be reached through replication. It is still possible to gain some additional confidence in the hypothesis by means of additional replications, but depending on the number of previously conducted experiments, it may be close to negligible. In light of that, what is the best research strategy to follow in the more realistic situation of limited resources?

Unfortunately, in the meta-research literature, there is no consensus concerning which kind of replication is most informative or which should be prioritized. For instance, some scholars, like Ioannidis, have argued that only direct replication can provide any support for the original hypothesis.

“However, they have a major drawback: almost anything can fit into a triangulation narrative by invoking some speculative “biological plausibility” as the connecting glue.

Most of these conceptual and triangulation links are likely overstated leaps of faith. Otherwise, it is very difficult to explain why we have so many successful narratives in the basic sciences, but very few of these proposed discoveries eventually work in humans. Moreover, a published conceptual replication with a different design and/or experimental conditions does not say how many laboratories have tried and how many different designs and experimental conditions failed and remain unpublished’’ (Ioannidis, 2017, p. 944).

While others have argued that conceptual replications should be preferred over direct replications.:

“We believe that this support is important and convincing because it was obtained in a different culture and with an entirely different ideology. In contrast to an exact replication, such a conceptual replication points to the validity of the underlying theory and suggests that an underlying mechanism of potentially universal application has been identified” (Roebroeck & Guimond, 2017).

A similar preference for conceptual replication guides the publishing policy adopted by some scientific journals. For example, editors of the International Journal of Research in Marketing discouraged direct replications and instead promoted conceptual ones. And this policy resulted in a high success rate (out of 30 published replication results, only 4 gave results that were less than very similar, which is a very high number considering the estimated replicability rates in the field, see Lynch et al., 2015). However, Ioannidis (2005) pointed out another important problem with conceptual replications: there is a great deal of flexibility in how to conduct them. Such flexibility was identified as one of the factors which decrease the reliability of an experiment and therefore the replicability of the result. Therefore, it may be the case that the high success rates of the conceptual replications published in IJRM may be due to all the bad reasons. Similarly, a meta-analysis of the studies replicated in large replication projects showed that an internal conceptual replication (conducted by the authors of the original result) does not increase the chance of a study being successfully directly replicated by independent researchers (Kunert, 2016). A successful direct replication is also a reliable indicator of the internal validity of the finding. For this reason, direct replications are more reliable in establishing the existence of the reported effect and therefore, we should start the effort to validate a given result with replications from this level.

In light of that, it seems to us that it is best to start with a direct replication. If it fails, it means that the result of the first experiment was likely to be a fluke and we no longer have any reason to have confidence in the result (Nosek et al., 2012). A failed direct replication suggests that the original hypothesis is false. Looking for a way to conceptually replicate a result, considering the failed direct replication or even in the absence of a successful one seems, as pointed out by Ioannidis, desperate or even unjustified. Vice versa, if a direct replication is successful, the next step is to try to support the hypothesis through an experiment that is different in some respects from the original one. For example, researchers may identify the weakest or most controversial part of the original experiment and try to replicate the hypothesis with an experiment that does not rely on this feature. The last step would be to conceptually replicate the hypothesis.^11^

### Which Results Should be Replicated? Direct Costs and Opportunity Costs

The problem of the costs of replication studies cannot be overlooked. In fact, replication studies have both direct costs (in terms of investment of time and energy) and opportunity costs (in terms of research that could otherwise have been done). Then, even if we were right and the epistemic usefulness of replications cannot be questioned, the conclusion can still be that replications should not necessarily be over-prioritized. Moreover, looking at the number of papers published per year versus the number of scientists, the ratio for 2021 is 3 million to 7–9 million. And in that population of scientists (which includes e.g., scientists doing research for private companies), only about 1% of them publish more than 1 article per year (Desmond, 2021). These are impressive numbers: so many studies are published, and there are not so many skilled and productive scientists around. Given the limited resources available it is obvious then that not all the studies can be replicated, and decisions need to be made about which articles are worth replicating.

As Desmond notes “Normative guidelines on replication also reflect this reality: a recent guideline explicitly recommends replication researchers to prioritize those assertions when the results from replication will have a major impact on scientific knowledge (KNAW 2018). Or to put it more crudely: do not bother with replicating insignificant assertions” (Desmond, 2021, p. 912). More recently, the National Academies of Science have published a consensus study report on replicability that also includes a “set of criteria to help determine when testing replicability may be warranted” (see National Academies of Science, 2019). Some of the items refer again to the scientific importance of the studies, while other items point to their methodological quality: we should prioritize the replication of “problematic” studies, whereas if a study is methodologically strong, its results should be believed. At the same time, Yarkoni (2019) convincingly argues that some results obtained in experiments of poor methodological quality are not generalizable and therefore not worth replicating. Given this, it seems that replication efforts should focus on important results that are not yet well-established but are supported by results obtained in well-designed studies.

What counts as a significant assertion depends on several considerations which are outside the scope of this paper. It is worth noting that assessing the importance of scientific findings is not an easy task, and it might lead to large disagreements among the members of the scientific community. It is easy to imagine that scientists would defend the importance of the results published in their area of research. Assessing the methodological quality of a scientific study might be much easier, as there is a large consensus on the possible biases and methodological devices to control for them. In certain fields, there are even specific tools to assess the risk of bias (see e.g., Berger & Exner, 1999).

In our view, the question of which studies are worth replicating remains open. As we argued, replication is always an epistemically beneficial activity, and it should be promoted and incentivized. At the same time, replications have costs, and resources in science are limited, so it is crucial to identify the studies worth replicating. This decision influences the most appropriate type of replication. In general, we suggested starting with direct replications as they are usually cheaper and easier to conduct. But if we are replicating a methodologically robust study, running a direct replication might be a waste of time and resources and we should rather move towards more conceptual replications. All in all, it seems that a sort of case-by-case cost–benefit analysis is always needed, that is, for each study, what we can learn should be assessed in light of expected costs.

## Conclusion

In this paper, we have proposed a defense of replicability as the cornerstone of experimental science, the "orthodox view," against the more cautious philosophical view towards it, "the new localism." We argued that all replications, like any other experiments, provide us with additional evidence and therefore are epistemically useful. Moreover, the additional epistemic importance of replicability can be appreciated according to the type of replication at stake. Finally, we have argued that replicability should be pursued in the case of all experimental results. Irreplicable results are not able to accommodate incoming evidence and therefore will no longer be empirically adequate as soon as new evidence is collected.

Our defense of the principle of replicability is motivated by a recent work of some influential philosophers of science, according to whom we should not talk about replicability as a hallmark of science. In addition to the problems, we discussed in the paper, their accounts often lack a discussion of the normative consequences for scientific practice. If we abandon replicability as a guiding principle of experimental research, what should scientists pursue instead? Most scientists agree that the reliability of scientific findings is important and strive to achieve replicable results, but what other mechanisms can warrant such reliability remains unclear. It would be desirable to have less costly or theoretically demanding alternatives to replicating experiments, but so far, we are not aware of any. Our argument has the merit of making sense of the emphasis on replication and the significant investment towards it. If philosophers of science are correct in their claim that replication is overrated, it is difficult to explain why the scientific community puts so much effort into it. Scientists may simply be mistaken—philosophers could respond. However, seeking to understand the epistemic activism toward replications of experiments seems to be a more fruitful and charitable approach to studying science.

## Footnotes

1 For example, see Irvine (2021): “In philosophical circles, direct replications are deemed to be largely uninformative about the theoretical claims an experiment is aimed at testing. (p. 847)’’ or “In fact, there is an emerging consensus in philosophy of measurement that even good conceptual replications do not offer a significant theoretical payoff, (p. 848)”.

2 This issue has been highlighted also by Leonelli (2018): “Given these issues, it is important to ask why direct reproducibility proves so attractive as an ideal to which research should aspire (p. 140)”.

3 In this section, we do not intend to present a normative proposal concerning how scientists should understand the replicability (for a promising normative account see e.g., Machery 2020; Nosek & Errington, 2020). Neither to perform a review of the literature (see e.g., Barba, 2018; Matarese, 2022).

4 The concept of replicability is important not only in the context of experimental results. Replicability can be also predicated on mathematical and computer-generated proofs or computational models (see e.g., Miłkowski et al., 2018). In the paper, we will restrict ourselves to discussing the concept of replicability as used in experimental science.

5 A similar argument was presented in (Penders Holbrook and de Rijcke, 2019).

6 “Direct replication is either extremely narrow in what it can deliver, or it runs into the joint problems of confounders and conceptual scope. Conceptual replication, on the other hand, cannot come to the rescue, because it is either begging the question or runs into similar problems” (Feest, 2019, p. 901).

7 “Instead, replication studies conducted in the absence of well-developed theory (and often in the presence of well-developed theory as well) are much better seen as exploratory studies that support different stages of theory development concerning a target phenomenon.”(Irvine, 2021, p. 850).

8 The assumption claiming that the designs of the original experiment and replication are similar is surprisingly hard to satisfy. For example, as described in Hensel (2020), failures in communication may make direct replication impossible. Replicating team has to know the details of the design of the original experiment and in many cases, such details are not available.

9 This approach is called triangulation (see e.g., Heesen, Bright, and Zucker, 2014).

10 This principle is often called the “variety of evidence thesis” (Hartmann and Bovens, 2001).

11 A similar sequential strategy or replication is presented in (Hüffmeier et al., 2015).
